# UPLC-MS/MS Determination of Twelve Ginsenosides in Shenfu Tang and Dushen Tang

**DOI:** 10.1155/2019/6217125

**Published:** 2019-07-11

**Authors:** Dawai Yang, Xiaofang Yang, Han Yan, Bin Fan, Jingang Dai, Jun Song, Yan Lei, Na Guo

**Affiliations:** ^1^Zhong Yuan Academy of Biological Medicine, Liaocheng People's Hospital, Liaocheng University, Liaocheng 252000, China; ^2^Experimental Research Center, China Academy of Chinese Medical Sciences, Beijing 100700, China

## Abstract

Shenfu Tang and Dushen Tang (one of the composite medicines for Shenfu Tang) are widely used Traditional Chinese herbal formulations and ginsenosides are their main bioactive components. However, there are rare studies about simultaneous analysis of ginsenosides in Shenfu Tang and Dushen Tang. In order to identify ginsenosides in Shenfu Tang and Dushen Tang and to explore law of compatibility of medicines in the decoction, a method for simultaneous determination of twelve ginsenosides in Shenfu Tang and Dushen Tang was developed by ultraresolution liquid chromatography coupled with tandem mass spectrometry (UPLC-MS/MS). The method showed satisfactory linearity (r > 0.9915), repeatability (RSD < 9.58%), intra- and interday precisions (RSD<11.90%), and high yields of recovery (92.26-113.20%) for twelve major constituents, namely, ginsenosides-Rb1, Rb2, Rb3, Rc, Rd, Rg1, Re, Rf, Rg2, Rg3, Rh1, and F2. Furthermore, the concentration of twelve ginsenosides in Dushen Tang and Shenfu Tang was also simultaneously analyzed. Most of ginsenosides except Rg1 and Rb1 showed higher contents in Shenfu Tang compared to Dushen Tang. The compatibility of the formula had the effect of promoting or inhibiting the dissolution of some major components. The present research provided a reliable evidence for the illustration of chemical basis and compatibility regularity of Shenfu Tang. This study demonstrated the utility of the developed method for assessment of the quantity of the major constituents in Dushen Tang and Shenfu Tang.

## 1. Introduction

Traditional Chinese herbal formulation has been widely used in the clinic for its well-proven efficacy with few side effects [1, 2]. Decoction is the traditional prescription of traditional Chinese medicines (TCM). One single herb or several kinds of herbs combined are boiled in water to make the decoction based on TCM theory [3]. Shenfu Tang, which was documented originally in 1465, is a famous TCM decoction with 3:2 ratio of Radix Ginseng and Fuzi (Radix Aconiti lateralis praeparata). It has been widely used to treat various diseases with signs of Yangqi decline or Yang exhaustion and especially for cardiovascular diseases [4–6]. Dushen Tang, which was traced back to 1600 years ago, is the decoction of the root of single Panax ginseng [3] and one of the composite medicines for Shenfu Tang. Ginseng is commonly used medicinal plant in both East Asia and the West and has been used as a tonic and a panacea that enhance physical performance and increase resistance to stress and aging [7, 8].

The main bioactive components of Dushen Tang and Shenfu Tang are ginsenosides since their major components are ginseng according to their formula. Ginsenosides have multiple pharmacological activities, including antioxidant, anti-inflammatory, antifatigue, and immunity-improving properties [9]. Therefore, it is necessary to investigate phytochemistry, metabolism, and quantity of ginsenosides in Dushen Tang and Shenfu Tang using modern technologies. To date, there were about 150 ginsenosides that were identified in the previous studies [10].

**Figure 1:**
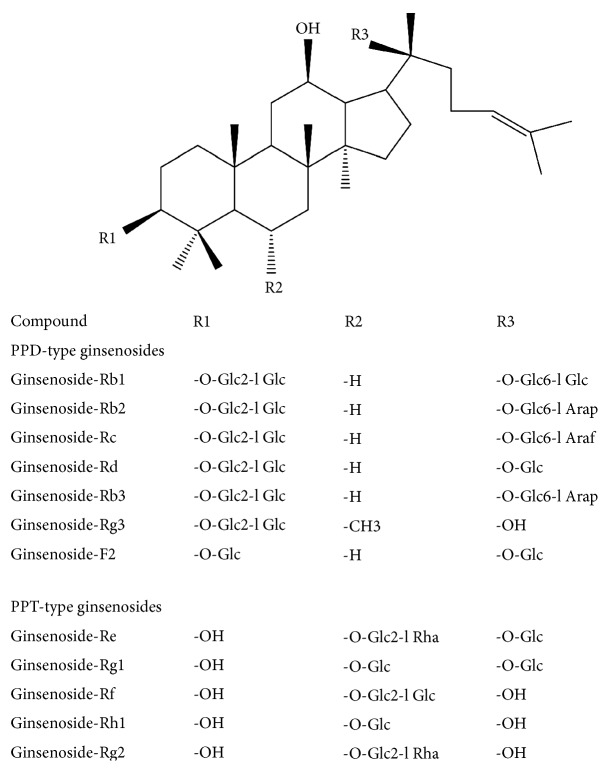
Chemical structures of ginsenosides.

Many techniques including HPLC [11–13], GC-MS [14, 15], and LC-MS [16–20] were used to analyze ginsenosides. Among them, the LC-MS and tandem mass spectrometry (MS/MS) techniques have been widely used for analysis of ginsenosides, because of their high dynamic range of detection, high sensitivity, and specificity [3, 21]. Many works have been carried out for the identification, quantification, and quality control of ginsenosides in raw plant materials, extracts, and marketed products [22–24]. Ginsenosides-Rb1, Rb2, Rc, Rd, Rg1, Re, and Rf (Figure 1) have been identified in Dushen Tang and Shenfu Tang in the previous studies [19, 25]. But simultaneous comparatively determination of twelve ginsenosides in Dushen Tang and Shenfu Tang has not been studied until now. Therefore, in order to further find out the prescriptions of traditional Chinese medicine, we confirmed major compounds in Dushen Tang and Shenfu Tang and determined them simultaneously by using UPLC-MS/MS.

The aim of the present study was to develop a direct and rapid LC-MS/MS method to simultaneously quantify twelve ginsenosides in Shenfu Tang and one of its composite medicines, Dushen Tang, namely, ginsenosides-Rb_1_, Rb_2_, Rb3, Rc, Rd, Rg_1_, Re, Rf, Rg2, Rg3, Rh1, and F2 (Figure 1). In addition, the concentration of twelve ginsenosides in Dushen Tang and Shenfu Tang was also determined.

## 2. Materials and Methods

### 2.1. Chemicals, Standards, and Samples

MS-grade acetonitrile and formic acid were purchased from Fisher Scientific (USA). All other chemicals and solvents were of an analytical grade. Ultrapure water (18.2AMΩ) was prepared with a Milli-Q water purification system (Millipore, Bedford, MA, USA). The reference standards of ginsenosides-Rb_1_, Rb_2_, Rb3, Rc, Rd, Rg_1_, Re, Rf, Rg2, Rg3, Rh1, and F2 were purchased from the National Institute for Control of Pharmaceutical and Biological Products (Beijing, China). The structures of these compounds are listed in Figure 1. The purity of the standards was no less than 98%. The commercial white ginseng samples were purchased from Jilin Shengyuan Changbai Mountain Pharmaceutical Co., Ltd. in China. The processed aconite root (Radix Aconiti lateralis praeparata) was purchased from Tong-Ren-Tang Pharmaceutical store (Beijing, China) and authenticated by Professor Xirong, HE, Institute of Traditional Chinese Medicine, China Academy of Chinese Medical Sciences.

### 2.2. Sample Preparation

#### 2.2.1. Reference Standards Solutions

Stock solutions: certain amounts of ginsenoside-Rb_1_, Rb_2_, Rb3, Rc, Rd, Rg_1_, Re, Rf, Rg2, Rg3, Rh1, and F2 were dissolved in methanol, respectively, to get reference standards solutions (1.0 mg/mL) and were stored below 4°C.

#### 2.2.2. Extracts of Shenfu Tang and Dushen Tang

Shenfu Tang is a TCM prescription with a 3:2 ratio of* Radix Ginseng *and Fuzi (*Radix Aconiti lateralis praeparata*). Dried and pulverised white ginseng (1200 g) and the processed aconite root (800 g) were ground and then refluxed three times with 20 L of water for 60 min at 100°C based on our published papers [19, 25]. After cooling, the extracting solutions were filtered and condensed under decompression and finally were freeze-dried. The extraction rate is 31.93%.

Dried and pulverised white ginseng (1000 g) were ground and then refluxed three times with 10 L of water for 60 min at 100°C. After cooling and filtering, the solutions were condensed under decompression and were finally freeze-dried. The extraction rate is 42.4%.

The decoction extracts were dissolved, respectively, in a certain amount of water to a concentration 10mg/mL. 0.5 mL of the solutions was precipitated, respectively, with 4 mL ethanol allowed to sit for 24 h at 4°C. The solutions were centrifuged at 4500 rpm for 10 min. After that, the solutions were diluted 50 times and centrifuged at 4000 r/min for 5 minutes.

### 2.3. Method Validation

An external calibration method was used for the quantitative analysis. The linearity calibration curves were constructed by six different concentrations of twelve ginsenosides. Each concentration was analyzed in triplicate and then the calibration curves were constructed by plotting the peak areas versus the concentrations of each analyte. The limit of detection (LOD) and limit of quantification (LOQ) were measured with the signal-to-noise ratio of 3 and 10, respectively, as criteria. The intraday precision was performed by analysis of the standard solution at six times within 1 day, while the interday precision was determined by repeated analysis of the sample for consecutive 3 days. For repeatability test, six independent sample solutions were prepared in the procedures noted. For stability test, the sample solutions set for three hours to 24 hours were analyzed. The recovery of this method was achieved using the standard addition method. The average recoveries were determined by the following formula: Recovery (%) = (Observed amount-Original amount)/Spiked amount×100 %, RSD (%) = (SD/mean)×100 %.

### 2.4. UPLC-MS Conditions

A Waters ACQUITY UPLC system (Milford, MA, USA), which was equipped with a Waters (Milford, MA, USA) Xevo TQ-S triple quadrupole mass spectrometer and operated in positive ion mode (data analysis software MassLynx™ V4.1), was used for simultaneous determination of twelve ginsenosides in Shenfu Tang. The separation was performed on ACQUITY UPLC BEH C18 (100 mm×2.1 mm, 1.8 *μ*m). The mobile phase consisted of (A) water containing 0.1% formic acid and (B) acetonitrile containing 0.1% formic acid. The linear gradient conditions were as follows: 0.0-1.5 min, 100.0-80.0 % B; 1.5-3.0 min, 80.0-73.0 %; 3.0-5.0 min, 73.0-73.0 %; 5.0-5.5 min, 73.0-72.0 %; 5.5-12.0 min, 72.0-70.0 %; 12.0-15.0 min, 70.0-69.0 %; 15.0-18.5 min, 69.0-50.0 %; 18.5-21.5 min, 50.0-40.0 %; 18.5-21.5 min, 50.0-40.0 %; and 21.5-22.0 min, 40.0-0.0 %. The flow rate was 0.30 ml/min. The column temperature was 45°C.

The conditions of MS analysis were as follows: the desolvation gas was set to 600 L/h at temperature of 400°C; the cone gas was set to 150 L/h, respectively. The capillary voltage and sampling cone voltage were set to 3500 V and 80 V, respectively.

## 3. Results and Discussion

### 3.1. Optimization of LC-MS/MS Conditions

**Figure 2:**
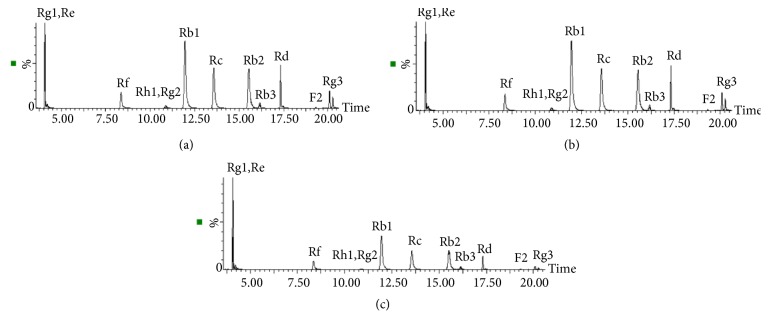
Typical UPLC-QQQ MS/MS chromatograms of twelve ginsenosides: (a) standard mixture, (b) Shenfu Tang, and (c) Dushen Tang.

In order to get best chromatographic resolution, many mobile phases including acetonitrile and methanol 0.05 %, 0.1 %, and 0.2 % aqueous formic acid and acetic acid were tested in the present study. The best peak shape and resolution were obtained from a mixture of (A) water containing 0.1% formic acid and (B) acetonitrile containing 0.1% formic acid. The typical LC-MS/MS chromatograms of ginsenosides in standard mixtures, Shenfu Tang and Dushen Tang, are shown in Figure 2. From Figure 2, we can see that ginsenosides were separately eluted within 22 min by optimizing elution gradient. Meanwhile, ionization mode, capillary voltage, fragmentor voltage, collision energy, gas flow, and desolvation temperature were optimized as well.

The optimized MS/MS method could achieve highest response using the multiple reaction monitoring (MRM) pairs consisting of the precursor and product ions. IntelliStart (Waters Acquity UPLC system built-in software) was used to optimize the mass conditions for the ginsenosides. The results of IntelliStart for the 12 ginsenosides (including cone voltage optimization, optimized MS spectrum, collision energy optimization, and optimized daughter spectrum) can be founded in Supplementary Materials. The optimized precursor and product ions of the twelve ginsenosides are shown in Table 1. The optimum collision energy was determined to be from 36 eV to 64 eV for different ginsenosides (Table 1).

### 3.2. Calibration Curves, LODs, and LOQs

The linearity of the developed method was assessed using six different concentrations of twelve ginsenosides. The standard calibration curves of all compounds were shown in Table 2 with satisfactory linearity (r > 0.9915). The LODs and LOQs of the twelve analytes were 0.30~8.00 and 0.80~20.00 ng/mL, respectively.

### 3.3. Precision, Repeatability, and Stability

The precision of the developed assay was evaluated with intraday and interday variations. The analyzed data showed that relative standard deviation (RSD) of intraday was in the range of 1.61-11.90% at six times within 1 day, and the RSD of interday was in the range of 2.43-22.24% determined by repeated analysis of the sample for consecutive 3 days (Table 3). The repeatability was satisfactory with RSD below 9.58% by testing six independent samples (Table 3). For stability test, the samples were analyzed at 3h, 6h, 9h, 12h, and 24h (Table S13). The RSD of stability test was in the range of 6.94-13.74%.

### 3.4. Accuracy

The accuracy of the method was assessed by a recovery assay. Before we did the test of recovery, the initial amount of 12 ginsenosides were tested firstly in accordance with the methods mentioned above, and the spiked samples were then extracted, processed, and quantified.  Table 4 showed that the recovery of the twelve ginsenosides ranged from 92.26% to 113.20%, and their RSD values were all less than 5.4%. Recovery data represented the accuracy of the method and is sufficient for further sample analysis.

### 3.5. Analysis of Ginsenosides in Dushen Tang and Shenfu Tang

**Figure 3:**
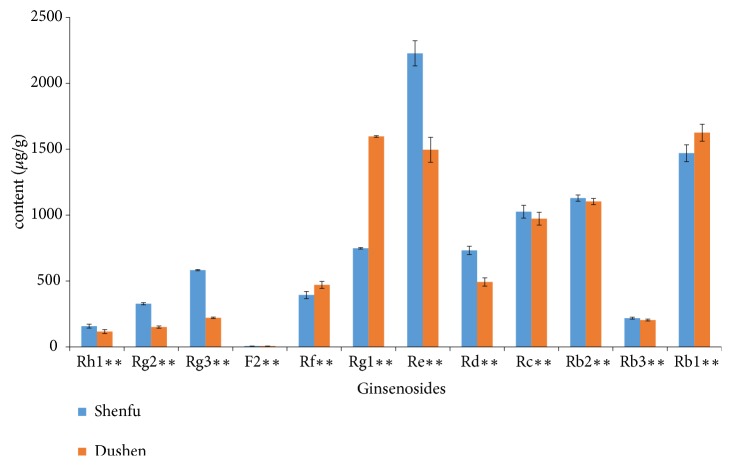
Concentrations of ginsenosides in Dushen Tang and Shenfu Tang. *∗∗*p<0.01.

The established analytical assay was subsequently applied for the simultaneous determination of the twelve ginsenosides in Dushen Tang and Shenfu Tang. From Figure 3, we can see that there are significant differences among the contents of the twelve ginsenosides between Dushen Tang and Shenfu Tang. The results showed most of ginsenosides; except Rg1 and Rb1 showed higher contents in Shenfu Tang compared to Dushen Tang according to crude botanicals per gram (Figure 3).

Composite formulae are one of the most important characteristics of TCM to obtain synergistic effects or to diminish possible adverse reactions. Usually, the therapeutic efficacy of herbal medicines is achieved by combinatorial components rather than single compound [26]. The curative effect of Shenfu Tang is an integrative result of a number of ginsenosides and alkaloids [19]. Therefore, the differences of twelve ginsenosides between Dushen Tang and Shenfu Tang could be explained by law of compatibility of medicines in Dushen Tang and Shenfu Tang. Further study for the changes of the active compounds in Dushen Tang and Shenfu Tang (that means before and after compatibility of ginseng) is needed to carry out.

## 4. Conclusions

In this study, a UPLC-MS/MS method for the simultaneous determination of twelve active ginsenosides in Dushen Tang and Shenfu Tang has been developed. The results showed that it could be used for the quality control of Dushen Tang and Shenfu Tang. In addition, compared with Dushen Tang, most of ginsenosides except Rg1 and Rb1 showed higher contents in Shenfu Tang; the different quantities of ginsenosides or active compounds in Dushen Tang and Shenfu Tang reflected the law of compatibility of medicines based on TCM theory.

## Figures and Tables

**Table 1:** Mass spectra properties of twelve ginsenosides.

Compounds	Precursor Ion	Product Ion	Dwell(s)	CV(V)	CE(V)
Rh1	661.5	203.1	0.163	100	36
Rg2	807.7	349.2	0.163	100	44
Rg3	807.7	365.1	0.163	94	44
F2	807.7	627.5	0.163	28	40
Rf	823.7	365.1	0.163	100	46
Rg1	823.7	643.5	0.330	100	38
Re	969.8	789.6	0.163	100	42
Rd	969.8	789.6	0.330	100	46
Rc	1101.8	335.2	0.330	98	62
Rb2	1101.9	335.2	0.163	100	60
Rb3	1101.8	789.6	0.163	100	48
Rb1	1131.9	365.2	0.330	98	64

**Table 2:** Calibration curves, LOD and LOQ for twelve ginsenosides.

Compound Name	Calibration curve	r^2^	Linear range (ng/mL)	LOD (ng/mL)	LOQ (ng/mL)
Rh1	Y=1.2549X-0.1531	0.9985	31.25-1000.00	8.00	20.00
Rg2	Y=3.0121X+4.5875	0.9995	25.00-800.00	4.00	12.00
Rg3	Y=5.7582X+44.9854	0.9993	62.50-2000.00	0.30	0.80
F2	Y=19.1291X+9.5143	0.9948	1.09-35.00	1.00	10.00
Rf	Y=13.2170X+30.8831	0.9997	62.50-2000.00	0.50	1.50
Rg1	Y=15.9033X-11.0447	0.9830	140.63-2250.00	1.00	3.00
Re	Y=3.1045X+73.7435	0.9981	140.63-4500.00	0.60	1.50
Rd	Y=9.4545X+64.9408	0.9974	46.88-1500.00	0.60	2.50
Rc	Y=18.2217X-97.4775	0.9993	78.13-2500.00	0.50	2.40
Rb2	Y=18.0760X-823.578	0.9888	109.38-3500.00	2.00	6.00
Rb3	Y=5.2107X+16.0536	0.9981	23.44-750.00	3.50	10.00
Rb1	Y=20.6874X-509.2980	0.9987	109.38-3500.00	0.80	2.40

**Table 3:** Precision and Repeatability for the twelve ginsenosides.

Ginsenosides	Interprecision RSD (%) (n=6)			Intraprecision RSD (%) (n=3)			Repeatability RSD (%) (n=6)
	L	M	H	L	M	H	
Rh1	8.59	10.33	2.00	16.62	9.88	10.36	9.16
Rg2	4.74	10.38	3.21	18.82	18.99	20.11	4.57
Rg3	2.94	3.33	2.12	5.03	8.81	6.00	3.76
F2	11.90	3.57	1.93	6.90	12.18	6.63	5.99
Rf	2.70	4.31	2.75	22.24	19.71	15.89	3.04
Rg1	3.96	4.16	3.97	4.65	5.22	3.17	6.29
Re	2.70	2.14	3.39	8.91	5.74	5.97	5.99
Rd	2.70	2.26	3.18	8.58	5.51	4.92	5.98
Rc	3.62	2.87	2.82	14.24	10.50	4.27	5.17
Rb2	3.86	3.33	1.78	14.79	10.66	2.43	3.91
Rb3	2.56	3.85	3.15	18.55	9.81	15.42	9.58
Rb1	3.49	3.01	1.61	14.51	9.42	10.41	4.55

**Table 4:** Recovery for the twelve ginsenosides (n=6).

Compounds	Initial amount (ng)	Added amount (ng)	Detected amount (ng)	Recovery (%)	RSD (%)
Rh1	109.04	110.00	210.53	92.26	5.40
Rg2	232.89	230.00	452.45	95.46	3.56
Rg3	401.41	410.00	840.59	107.12	1.65
F2	3.90	3.50	7.86	113.20	4.25
Rf	264.02	270.00	528.47	97.95	3.03
Rg1	493.58	520.00	1064.54	109.80	3.62
Re	1458.43	1550.00	3162.58	109.95	3.33
Rd	479.99	510.00	1040.27	109.86	3.30
Rc	684.71	710.00	1462.20	109.51	2.24
Rb2	769.51	790.00	1524.60	95.58	1.05
Rb3	152.45	150.00	296.00	95.70	2.45
Rb1	997.10	1000.00	2085.35	108.82	1.10

## Data Availability

The data used to support the findings of this study are available from the corresponding author upon request.

## Supplementary Materials

Supplementary Materials Table S1. IntelliStart for Ginsenoside Rh1 Figure S1. A: Collision Energy Optimization (m/z 661.50→203.12); B: Optimized Daughter Spectrum (at collision Energy 36eV) Table S2. IntelliStart for Ginsenoside Rg2 Figure S2. A: Collision Energy Optimization (m/z 807.66→349.20); B: Optimized Daughter Spectrum (at collision Energy 44eV) Table S3. IntelliStart for Ginsenoside Rg3 Figure S3. A: Collision Energy Optimization (m/z 807.66→365.13); B: Optimized Daughter Spectrum (at collision Energy 44eV) Table S4. IntelliStart for Ginsenoside F2 Figure S4. A: Collision Energy Optimization (m/z 807.66→627.54); B: Optimized Daughter Spectrum (at collision Energy 40eV) Table S5. IntelliStart for Ginsenoside Rf Figure S5. A: Collision Energy Optimization (m/z 823.66→365.14); B: Optimized Daughter Spectrum (at collision Energy 46eV) Table S6. IntelliStart for Ginsenoside Rg1 Figure S6. A: Collision Energy Optimization (m/z 823.66→643.54); B: Optimized Daughter Spectrum (at collision Energy 38eV) Table S7. IntelliStart for Ginsenoside Re Figure S7. A: Collision Energy Optimization (m/z 969.76→789.60); B: Optimized Daughter Spectrum (at collision Energy 42eV) Table S8. IntelliStart for Ginsenoside Rd Figure S8. A: Collision Energy Optimization (m/z 969.76→789.60); B: Optimized Daughter Spectrum (at collision Energy 46eV) Table S9. IntelliStart for Ginsenoside Rc Figure S9. A: Collision Energy Optimization (m/z 1101.82→335.15); B: Optimized Daughter Spectrum (at collision Energy 62eV) Table S10. IntelliStart for Ginsenoside Rb2 Figure S10. A: Collision Energy Optimization (m/z 1101.89→335.23); B: Optimized Daughter Spectrum (at collision Energy 60eV) Table S11. IntelliStart for Ginsenoside Rb3 Figure S11. A: Collision Energy Optimization (m/z 1101.82→789.59); B: Optimized Daughter Spectrum (at collision Energy 48eV) Table S12. IntelliStart for Ginsenoside Rb1 Figure S12. A: Collision Energy Optimization (m/z 1131.89→365.20); B: Optimized Daughter Spectrum (at collision Energy 64eV) Table S13 Stability of twelve ginsenosides.
