# Supplementary material for: UPLC-MS/MS Determination of Twelve Ginsenosides in Shenfu Tang and Dushen Tang
Source: Int J Anal Chem. 2019 Jul 11;2019:6217125. doi: 10.1155/2019/6217125 (PMC6662505; doi:10.1155/2019/6217125)
Supplement: Supplementary Materials — Table S1. IntelliStart for Ginsenoside Rh1 Figure S1. A: Collision Energy Optimization (m/z 661.50→203.12); B: Optimized Daughter Spectrum (at collision Energy 36eV) Table S2. IntelliStart for Ginsenoside Rg2 Figure S2. A: Collision Energy Optimization (m/z 807.66→349.20); B: Optimized Daughter Spectrum (at collision Energy 44eV) Table S3. IntelliStart for Ginsenoside Rg3 Figure S3. A: Collision Energy Optimization (m/z 807.66→365.13); B: Optimized Daughter Spectrum (at collision Energy 44eV) Table S4. IntelliStart for Ginsenoside F2 Figure S4. A: Collision Energy Optimization (m/z 807.66→627.54); B: Optimized Daughter Spectrum (at collision Energy 40eV) Table S5. IntelliStart for Ginsenoside Rf Figure S5. A: Collision Energy Optimization (m/z 823.66→365.14); B: Optimized Daughter Spectrum (at collision Energy 46eV) Table S6. IntelliStart for Ginsenoside Rg1 Figure S6. A: Collision Energy Optimization (m/z 823.66→643.54); B: Optimized Daughter Spectrum (at collision Energy 38eV) Table S7. IntelliStart for Ginsenoside Re Figure S7. A: Collision Energy Optimization (m/z 969.76→789.60); B: Optimized Daughter Spectrum (at collision Energy 42eV) Table S8. IntelliStart for Ginsenoside Rd Figure S8. A: Collision Energy Optimization (m/z 969.76→789.60); B: Optimized Daughter Spectrum (at collision Energy 46eV) Table S9. IntelliStart for Ginsenoside Rc Figure S9. A: Collision Energy Optimization (m/z 1101.82→335.15); B: Optimized Daughter Spectrum (at collision Energy 62eV) Table S10. IntelliStart for Ginsenoside Rb2 Figure S10. A: Collision Energy Optimization (m/z 1101.89→335.23); B: Optimized Daughter Spectrum (at collision Energy 60eV) Table S11. IntelliStart for Ginsenoside Rb3 Figure S11. A: Collision Energy Optimization (m/z 1101.82→789.59); B: Optimized Daughter Spectrum (at collision Energy 48eV) Table S12. IntelliStart for Ginsenoside Rb1 Figure S12. A: Collision Energy Optimization (m/z 1131.89→365.20); B: Optimized Daughter Spectrum (at collision Energy 64eV) Tab [file 6217125.f1.doc]

# UPLC-MS/MS Determination of Twelve Ginsenosides in Shenfu Tang and Dushen Tang

Dawai Yang,^1^Xiaofang Yang,^2^ Han Yan,^2^ Bin Fan,^2^ Jingang Dai,^2^ Jun Song,^2^ Yan Lei^2^ and Na Guo^2^

^1^ Zhong Yuan Academy of Biological Medicine, Liaocheng People’s Hospital, Liaocheng 252000, P.R. China.
^2^ State Key Laboratory of Dao-di Herbs (preparing), Experimental Research Center, China Academy of Chinese Medical Sciences, Beijing 100700, P.R. China.

Correspondence should be addressed to Yan Lei; [13651217893@163.com](mailto:13651217893@163.com); Na Guo; [guona5246@126.com](mailto:guona5246@126.com).

Table S1. Intellistart for Ginsenoside Rh1

| Compounds | Formula | Parent m/z | CV | Daughters | CE |
| --- | --- | --- | --- | --- | --- |
| Ginsenoside Rh1 | 638.40 | 661.50 | 100 | 203.12 | 36 |


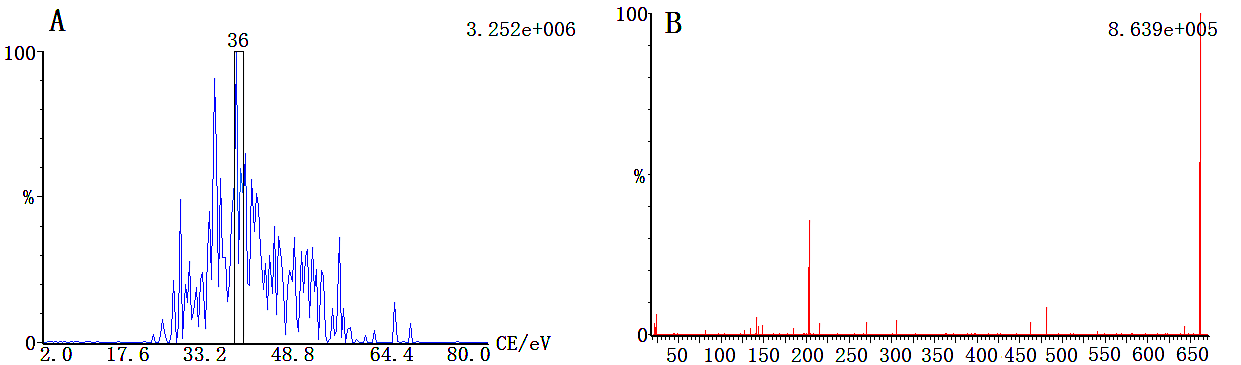


**Figure S1.** A: Collision Energy Optimization (m/z 661.50→203.12); B: Optimized Daughter Spectrum (at collision Energy 36eV)

Table S2. Intellistart for Ginsenoside Rg2

| Compounds | Formula | Parent m/z | CV | Daughters | CE |
| --- | --- | --- | --- | --- | --- |
| Ginsenoside Rg2 | 784.50 | 807.66 | 100 | 349.20 | 44 |


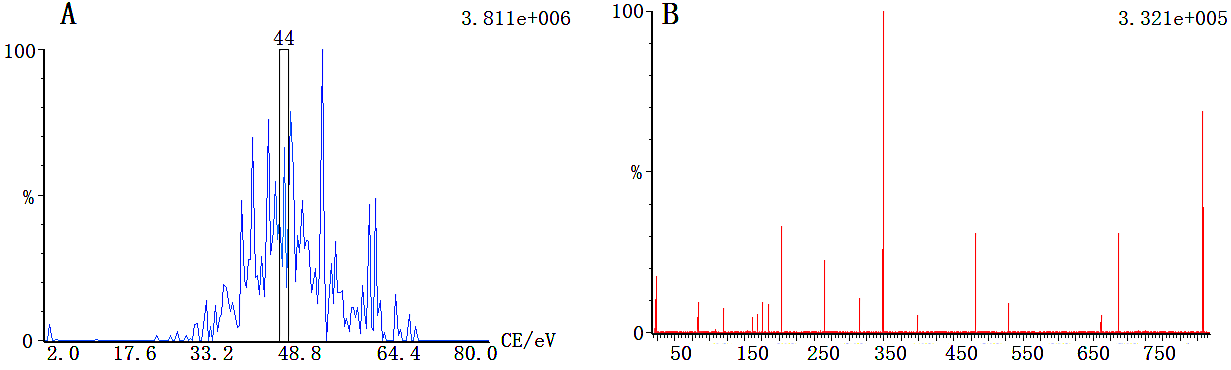


**Figure S2.** A: Collision Energy Optimization (m/z 807.66→349.20); B: Optimized Daughter Spectrum (at collision Energy 44eV)

Table S3. Intellistart for Ginsenoside Rg3

| Compounds | Formula | Parent m/z | CV | Daughters | CE |
| --- | --- | --- | --- | --- | --- |
| Ginsenoside Rg3 | 784.50 | 807.66 | 94 | 365.13 | 44 |


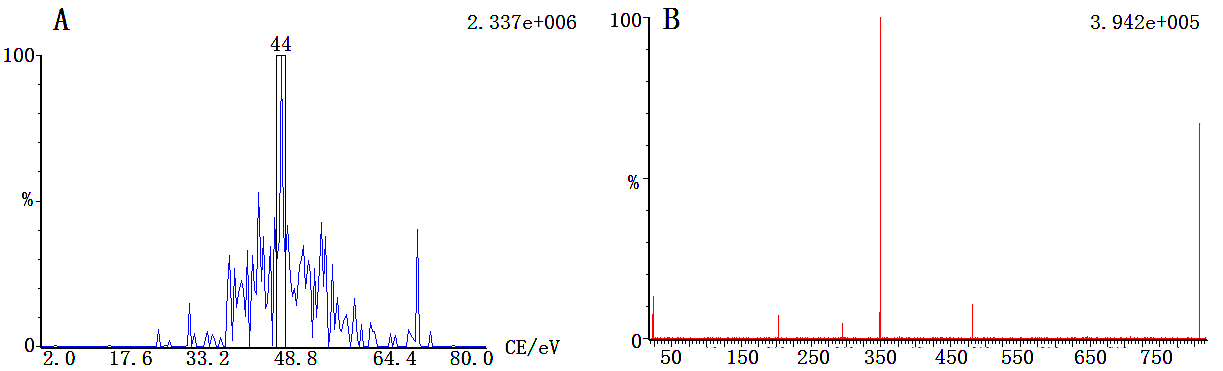


**Figure S3.** A: Collision Energy Optimization (m/z 807.66→365.13); B: Optimized Daughter Spectrum (at collision Energy 44eV)

Table S4. Intellistart for Ginsenoside F2

| Compounds | Formula | Parent m/z | CV | Daughters | CE |
| --- | --- | --- | --- | --- | --- |
| Ginsenoside F2 | 784.50 | 807.66 | 28 | 627.54 | 40 |

**
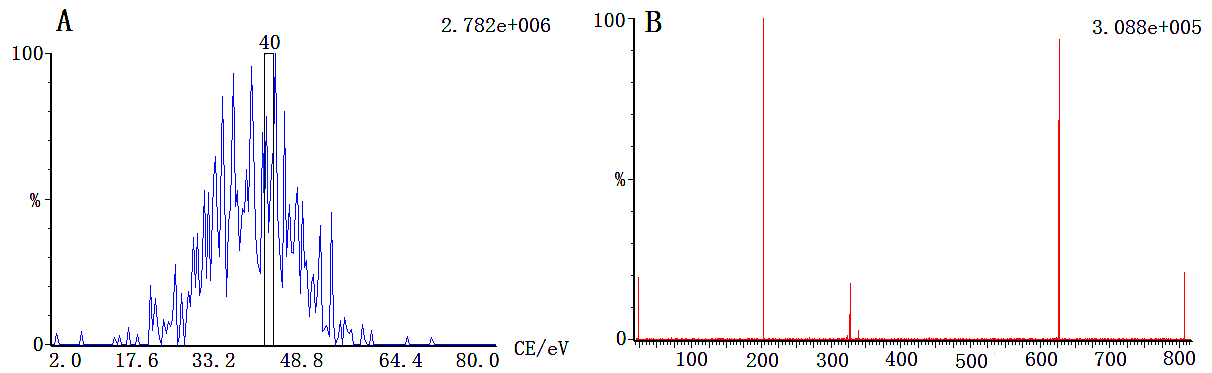
**

**Figure S4.** A: Collision Energy Optimization (m/z 807.66→627.54); B: Optimized Daughter Spectrum (at collision Energy 40eV)

Table S5. Intellistart for Ginsenoside Rf

| Compounds | Formula | Parent m/z | CV | Daughters | CE |
| --- | --- | --- | --- | --- | --- |
| Ginsenoside Rf | 800.50 | 823.66 | 100 | 365.14 | 46 |


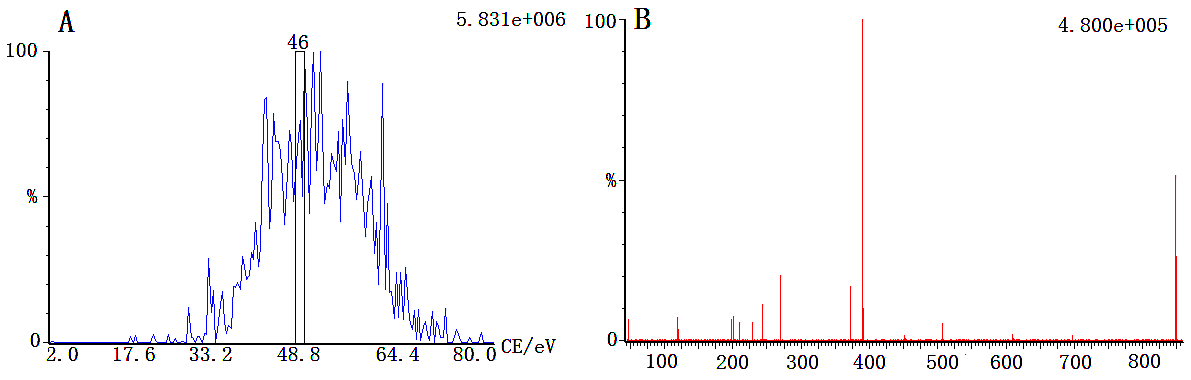


**Figure S5.** A: Collision Energy Optimization (m/z 823.66→365.14); B: Optimized Daughter Spectrum (at collision Energy 46eV)

Table S6. Intellistart for Ginsenoside Rg1

| Compounds | Formula | Parent m/z | CV | Daughters | CE |
| --- | --- | --- | --- | --- | --- |
| Ginsenoside Rg1 | 800.50 | 823.66 | 100 | 643.54 | 38 |


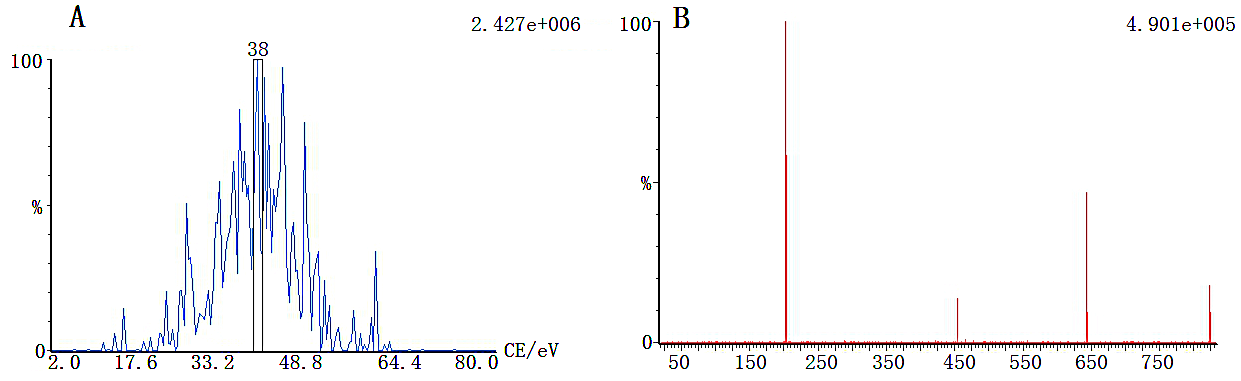


**Figure S6.** A: Collision Energy Optimization (m/z 823.66→643.54); B: Optimized Daughter Spectrum (at collision Energy 38eV)

Table S7. Intellistart for Ginsenoside Re

| Compounds | Formula | Parent m/z | CV | Daughters | CE |
| --- | --- | --- | --- | --- | --- |
| Ginsenoside Re | 946.60 | 969.76 | 100 | 789.60 | 42 |


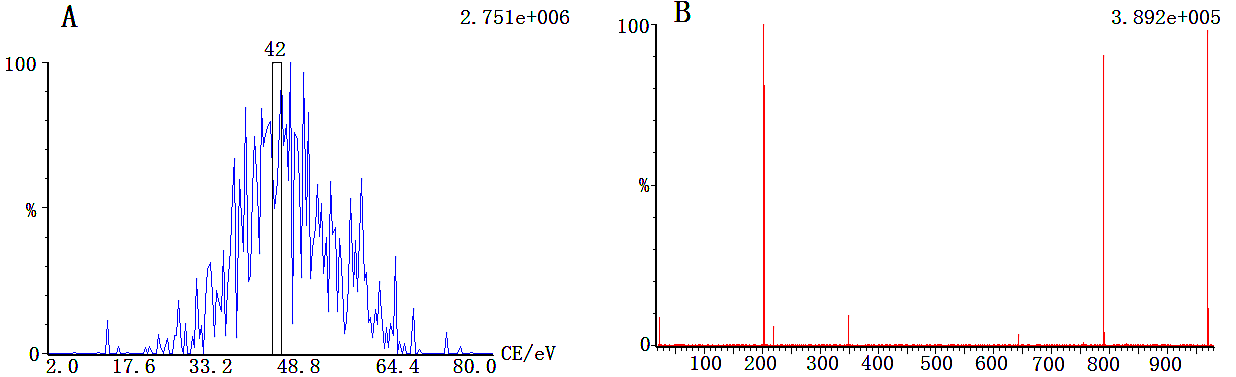


**Figure S7.** A: Collision Energy Optimization (m/z 969.76→789.60); B: Optimized Daughter Spectrum (at collision Energy 42eV)

Table S8. Intellistart for Ginsenoside Rd

| Compounds | Formula | Parent m/z | CV | Daughters | CE |
| --- | --- | --- | --- | --- | --- |
| Ginsenoside Rd | 946.60 | 969.76 | 100 | 789.60 | 46 |


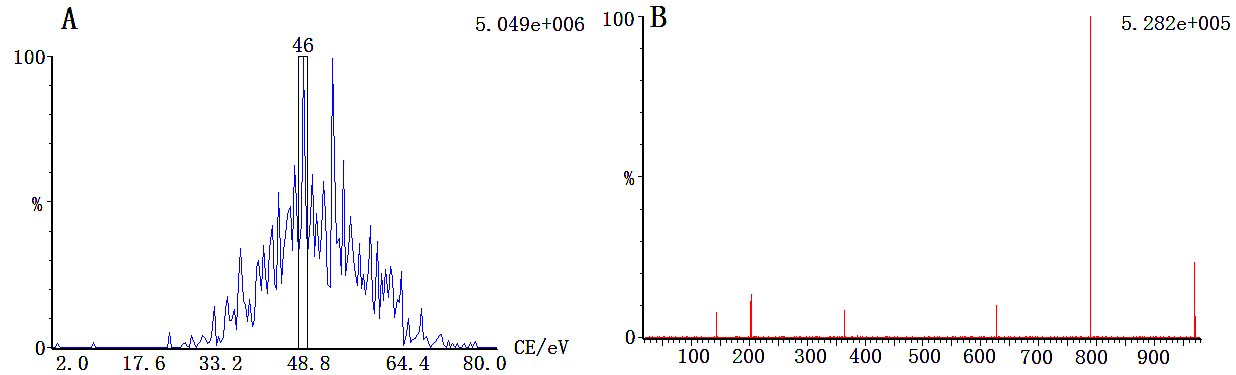


**Figure S8.** A: Collision Energy Optimization (m/z 969.76→789.60); B: Optimized Daughter Spectrum (at collision Energy 46eV)

Table S9. Intellistart for Ginsenoside Rc

| Compounds | Formula | Parent m/z | CV | Daughters | CE |
| --- | --- | --- | --- | --- | --- |
| Ginsenoside Rc | 1078.60 | 1101.82 | 98 | 335.15 | 62 |


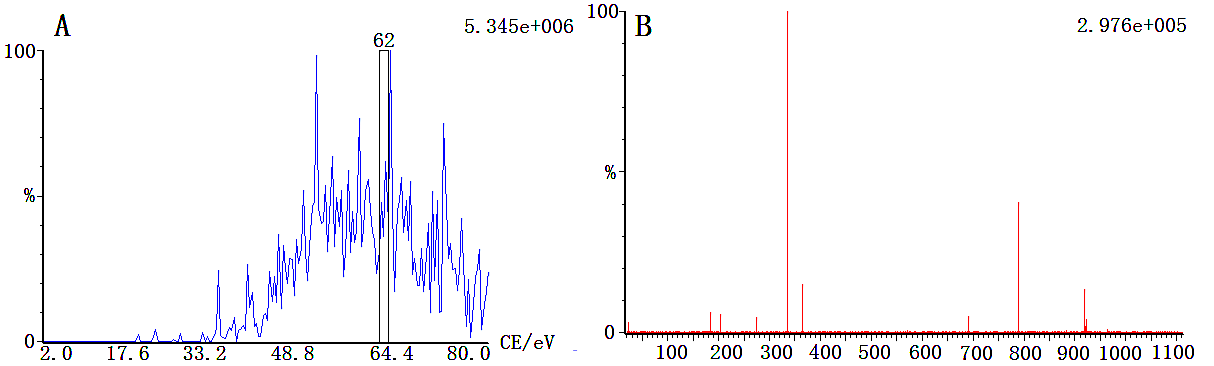


**Figure S9.** A: Collision Energy Optimization (m/z 1101.82→335.15); B: Optimized Daughter Spectrum (at collision Energy 62eV)

Table S10. Intellistart for Ginsenoside Rb2

| Compounds | Formula | Parent m/z | CV | Daughters | CE |
| --- | --- | --- | --- | --- | --- |
| Ginsenoside Rb2 | 1078.60 | 1101.89 | 100 | 335.23 | 60 |


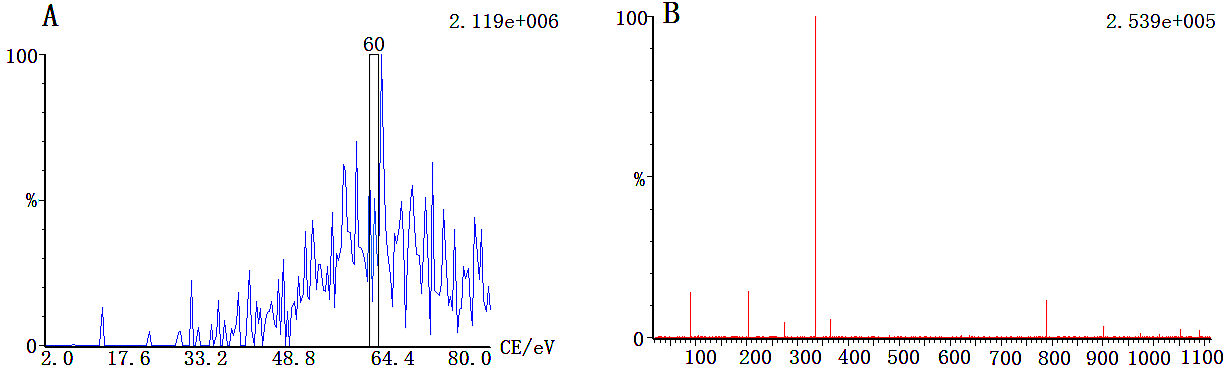


**Figure S10.** A: Collision Energy Optimization (m/z 1101.89→335.23); B: Optimized Daughter Spectrum (at collision Energy 60eV)

Table S11. Intellistart for Ginsenoside Rb3

| Compounds | Formula | Parent m/z | CV | Daughters | CE |
| --- | --- | --- | --- | --- | --- |
| Ginsenoside Rb3 | 1078.60 | 1101.82 | 100 | 789.59 | 48 |


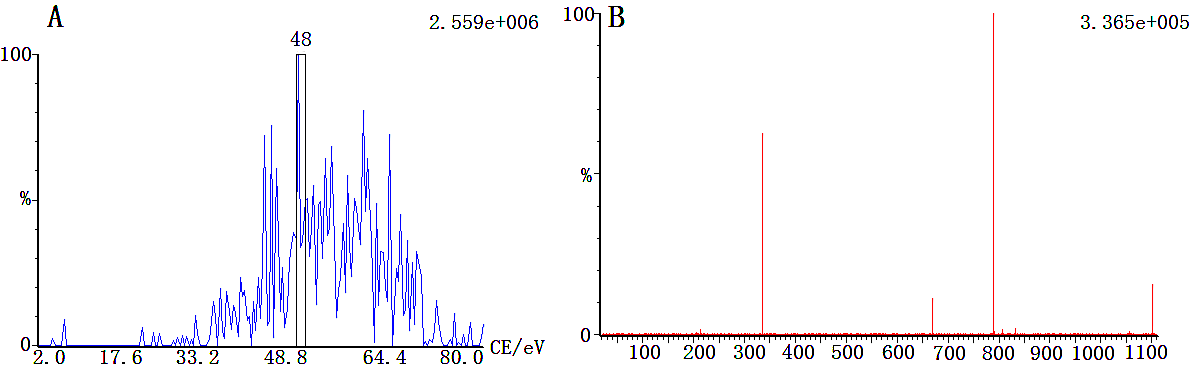


**Figure S11.** A: Collision Energy Optimization (m/z 1101.82→789.59); B: Optimized Daughter Spectrum (at collision Energy 48eV)

Table S12. Intellistart for Ginsenoside Rb1

| Compounds | Formula | Parent m/z | CV | Daughters | CE |
| --- | --- | --- | --- | --- | --- |
| Ginsenoside Rb1 | 1108.60 | 1131.89 | 98 | 365.20 | 64 |


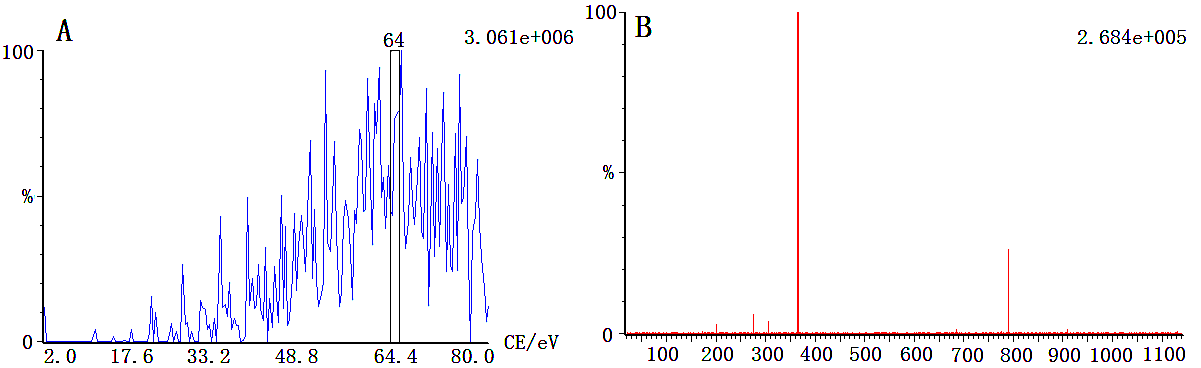


**Figure S12.** A: Collision Energy Optimization (m/z 1131.89→365.20); B: Optimized Daughter Spectrum (at collision Energy 64eV)

Table S13 Stability of twelve ginsenosides.

| Ginsenosides | 3 h  (ng/mL) | 6 h  (ng/mL) | 9 h  (ng/mL) | 12 h  (ng/mL) | 24 h  (ng/mL) | RSD (%) |
| --- | --- | --- | --- | --- | --- | --- |
| Rh1 | 111.33 | 101.76 | 106.95 | 121.23 | 128.55 | 9.52 |
| Rg2 | 244.24 | 216.77 | 230.65 | 272.75 | 285.50 | 11.47 |
| Rg3 | 433.49 | 395.70 | 404.19 | 460.19 | 492.18 | 9.13 |
| F2 | 3.51 | 3.36 | 3.63 | 3.76 | 4.68 | 13.74 |
| Rf | 285.29 | 263.71 | 256.69 | 305.85 | 303.48 | 7.92 |
| Rg1 | 562.34 | 523.97 | 458.18 | 528.84 | 551.96 | 7.74 |
| Re | 1654.84 | 1579.04 | 1438.93 | 1672.12 | 1742.28 | 7.14 |
| Rd | 544.32 | 519.87 | 473.48 | 550.47 | 573.65 | 7.15 |
| Rc | 776.44 | 694.73 | 684.74 | 789.51 | 798.00 | 7.28 |
| Rb2 | 843.98 | 762.64 | 774.05 | 854.28 | 898.98 | 6.94 |
| Rb3 | 178.93 | 151.02 | 148.71 | 169.84 | 160.25 | 7.87 |
| Rb1 | 1114.34 | 988.93 | 992.38 | 1117.58 | 1186.81 | 8.01 |
